# Evolutionary site-number changes of ribosomal DNA loci during speciation: complex scenarios of ancestral and more recent polyploid events

**DOI:** 10.1093/aobpla/plv135

**Published:** 2015-11-17

**Authors:** Marcela Rosato, Juan C. Moreno-Saiz, José A. Galián, Josep A. Rosselló

**Affiliations:** 1 Jardín Botánico, ICBiBE-Unidad Asociada CSIC, Universidad de Valencia, c/Quart 80, E-46008 Valencia, Spain; 2 Departamento de Biología, Facultad de Ciencias, Universidad Autónoma de Madrid, E-28049 Madrid, Spain; 3 Carl Faust Fdn., PO Box 112, E-17300 Blanes, Spain

**Keywords:** Brassicaceae, chromosome evolution, FISH, polyploidy, 5S rDNA, 45S rDNA, rDNA locus evolution, Vellinae

## Abstract

Several genome duplications have been identified in the evolution of seed plants, providing unique systems for studying karyological processes promoting diversification and speciation. Knowledge about the number of ribosomal DNA (rDNA) loci, together with their chromosomal distribution and structure, provides clues about organismal and molecular evolution at various phylogenetic levels. In this work, we aim to elucidate the evolutionary dynamics of karyological and rDNA site-number variation in all known taxa of subtribe Vellinae, showing a complex scenario of ancestral and more recent polyploid events. Specifically, we aim to infer the ancestral chromosome numbers and patterns of chromosome number variation, assess patterns of variation of both 45S and 5S rDNA families, trends in site-number change of rDNA loci within homoploid and polyploid series, and reconstruct the evolutionary history of rDNA site number using a phylogenetic hypothesis as a framework. The best-fitting model of chromosome number evolution with a high likelihood score suggests that the Vellinae core showing *x* = 17 chromosomes arose by duplication events from a recent *x* = 8 ancestor. Our survey suggests more complex patterns of polyploid evolution than previously noted for Vellinae. High polyploidization events (6x, 8x) arose independently in the basal clade *Vella castrilensis*–*V. lucentina*, where extant diploid species are unknown. Reconstruction of ancestral rDNA states in Vellinae supports the inference that the ancestral number of loci in the subtribe was two for each multigene family, suggesting that an overall tendency towards a net loss of 5S rDNA loci occurred during the splitting of Vellinae ancestors from the remaining Brassiceae lineages. A contrasting pattern for rDNA site change in both paleopolyploid and neopolyploid species was linked to diversification of Vellinae lineages. This suggests dynamic and independent changes in rDNA site number during speciation processes and a significant lack of correlation between 45S and 5S rDNA evolutionary pathways.

## Abstract (precis)

Genes encoding ribosomal RNA are universal key constituents of eukaryotic genomes, but the number of loci varies between species. We assessed the evolutionary trends in site-number changes of rDNA loci during speciation in a lineage of the cabbage family, characterized by complex scenarios of polyploidy. Our results suggest the existence of constrictions to burst loci amplification in the 5S rDNA family in polyploids and an overall trend to further reduce their number. The 45S rDNA site change in polyploids tells a different story, implying loci amplification in most of the polyploid entities.

## Introduction

Angiosperms have a long and complex history of whole-genome duplication (WGD) rounds since their origin. Comprehensive phylogenomic analyses of sequenced plant genomes, including molecular dating and phylogenetics, suggest a paleoploid event in the common ancestor of extant seed plants (ζ-WGD) and other in the common ancestor of extant angiosperms (ϵ-WGD) (Jiao *et al*. 2011). In addition, all core eudicots probably shared a hexaploidization event (γ-WGD) detected to date in asterids and rosids (*Arabidopsis thaliana*, *Carica papaya*, *Populus trichocarpa*, *Cucumis sativus* and *Vitis vinifera*; Amborella Genome Project 2013).

Moreover, additional and more recent paleopolyploidy events characterize some plant lineages. This is the case of Brassicaceae, the mustard family, where two more polyploidization events (α-WGD and β-WGD) have been identified in *Arabidopsis* and are shared with other members of the order Brassicales (Bowers *et al*. 2003). Other species from tribe Brassiceae have undergone a further whole-genome triplication since their divergence from *Arabidopsis*, an event dated between 13 and 17 Mya (Town *et al*. 2006) or perhaps as early as 43 Mya (Beilstein *et al*. 2010).

Within Brassicaceae, subsequent WGD events have occurred in different lineages including the tribe Brassiceae (Warwick and Al-Shehbaz 2006; Franzke *et al.* 2011). Thus, chromosome numbers as high as ∼240 have been reported in some genera (*Cardamine*; Warwick and Al-Shehbaz 2006), suggesting more complex scenarios of genome duplications at shallower evolutionary levels.

**Figure 1.**
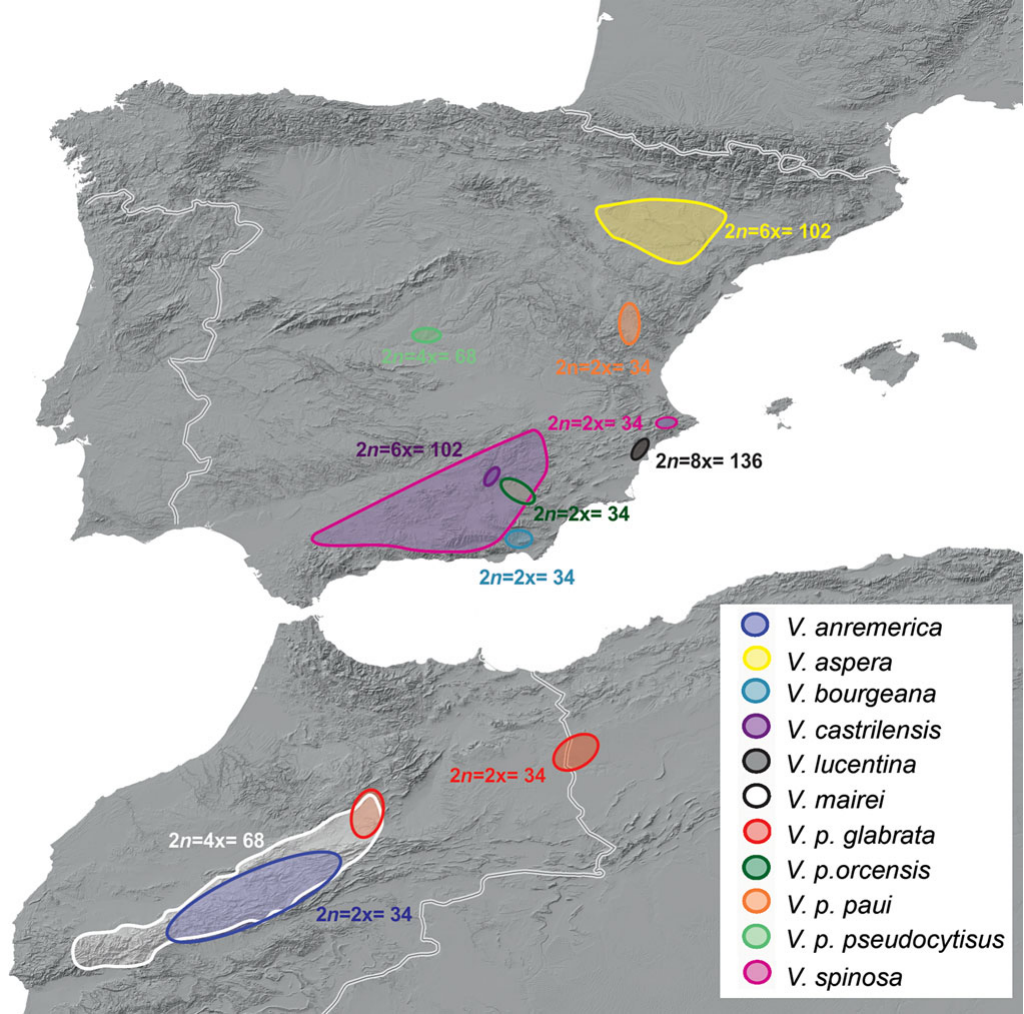
Geographical distribution of the genus *Vella*. Chromosome numbers found in this study and inferred ploidy levels are also indicated.

Brassiceae is one of the 25 recognized tribes in the family on the basis of morphological and molecular studies (Al-Shehbaz *et al*. 2006; Warwick *et al.* 2010). This tribe is a monophyletic assemblage including ∼51 genera and ∼240 species, which are consistently distinguished from the rest of the family by the presence of conduplicate cotyledons, transversely segmented fruits, and simple hairs when present (Warwick and Sauder 2005). Within Brassiceae, one of the few groups whose monophyly has received strong support is subtribe Vellinae (Warwick and Sauder 2005). This includes 3 genera (*Succowia*, *Carrichtera* and *Vella*) that altogether encompass 10 species and 3 additional subspecies (Crespo 1992; Warwick and Al-Shehbaz 1998; Crespo *et al*. 2005; Simón-Porcar *et al*. 2015), which are mainly distributed in the Western Mediterranean basin (Fig. 1).

Despite the low biodiversity currently found in subtribe Vellinae, a remarkable karyological diversity has been reported. Putative low chromosome base numbers are present in both annual basal genera of the subtribe, *Succowia* (*x* = 9) and *Carrichtera* (*x* = 8); in contrast, a higher, autopomorphic chromosome base number (*x* = 17) characterizes *Vella* (Warwick and Al-Shehbaz 2006). Putative diploid (2*n* = 34), tetraploid (2*n* = 68) and hexaploid (2*n* = 102) levels have been reported in *Vella* (Warwick and Al-Shehbaz 2006). These karyological features made subtribe Vellinae an adequate case study to address the fate and evolutionary dynamics of ribosomal DNA (rDNA) expansion and contraction in a complex scenario of several WGD events along different time periods of their evolutionary history.

Genes encoding rDNA are universal key constituents of the eukaryotic genomes as their products form the backbones of the functional cytoplasmic, plastid and mitochondrial ribosomes. In contrast with the single or low-copy number of rDNA genes present in the plastidial and mitochondrial genomes, the nuclear genome harbours hundreds to thousands of copies of each ribosomal species (18S, 5.8S, 25S/26S and 5S) that are usually arranged in distinct arrays of tandemly repeated units. Nuclear rDNA has been long regarded as merely involved in the biogenesis of both ribosomes and nucleolus (Hemleben and Werts 1988; Shaw and Jordan 1995). Recent evidence has dramatically changed this perception, suggesting that it plays additional roles in the biology of the cell, acting to preserve genome stability and trigger ageing (Kobayashi 2008).

Knowledge about the number of rDNA loci, together with their chromosomal distribution and structure, provides clues about organismal and molecular evolution at various phylogenetic levels. In this work, we aim to elucidate the evolutionary dynamics of karyological and rDNA site-number variation in all known taxa of subtribe Vellinae. Specifically, we aim to infer the ancestral chromosome numbers and patterns of chromosome number variation, assess patterns of variation of both 45S and 5S rDNA families, trends in site-number change of rDNA loci within homoploid and polyploid series and reconstruct the evolutionary history of rDNA site number using a phylogenetic hypothesis as a framework.

## Methods

### Plant material

Sampling was extensive and covered all 13 taxa included in subtribe Vellinae (Table 1).

**Table 1.** Origin of the samples used in the karyological study and GenBank accession numbers for the rDNA ITS sequences used in the phylogenetic study. Samples were collected in the field by the authors or received from seed bank accessions (codes are provided).

Species	Accession (seed bank code)	ITS
*Succowia balearica* (L.) Medik.	Balearic Islands, Mallorca, Felanitx	AF263395
*Carrichtera annua* (L.) DC.	Spain, Valencia, San Antonio de Benageber	DQ249829
*Vella anremerica* (Litard. & Maire) Gómez-Campo	Morocco, near Lake Tislit, Imilchil, Er Rachidia	AF263387
*V. aspera* Pers.	Spain, Lleida, Candasnos (Genmedoc 1438)	AF263394
*V. bourgeana* (Coss. ex Webb) Warwick & Al-Shehbaz	Spain, Almería, Tabernas (BGVA 13269-90)	AF263385
*V. castrilensis* Vivero *et al.*	Spain, Cazorla, Cabrilla Baja	AJ841702
*V. lucentina* M.B. Crespo	Spain, Alicante, Mutxamel (CIEF A139J)	AF263389
	Spain, Alicante, Monforte del Cid (CIEF A139K)	
*V. mairei* Humbert ex Maire	Morocco, High Atlas between Agoudal and Tizi-n-Ouano	AF263388
*V. pseudocytisus* L.		
subsp. *pseudocytisus*	Spain, Madrid, Aranjuez	AF263393
subsp. *glabrata* Greuter	Morocco, between Zeïda and Midelt	AF263392
subsp. *orcensis* Vivero *et al.*	Spain, Granada, Puebla de Don Fadrique	KT852986
subsp*. paui* Gómez-Campo	Spain, Teruel, Villel	AF263391
*V. spinosa* Boiss.	Spain, Alicante, Sierra Aitana (CIEF A582/A186A)	AF263390

### Chromosome preparations

Root tips from seeds germinated in agar plates (2.0 %) were removed, pretreated with 2 mM 8-hydroxyquinoline for 2 h at 4 °C, fixed in 3 : 1 ethanol : acetic acid (v : v) and stored at −20 °C until used. Root tips and flower buds were washed in 10 mM citrate buffer, pH 4.6, and then macerated in a mixture of 2 % (v/v) cellulase (Calbiochem) in citrate buffer, pH 4.6, and 2 % pectinase (from *Aspergillus niger*) in 40 % glycerol in 10 mM citrate buffer, pH 4.6, for 60 min at 37 °C. The meristematic cells were squashed and air-dried after coverslip removal by the dry-ice method. In the case of *V. lucentina*, immature inflorescences were directly fixed in an ethanol–glacial acetic acid (3 : 1) mixture and stored at 4 °C until required to study the male meiotic divisions according to the protocol described above. AgNO_3_ staining using silver impregnation was carried out on 1–2 day-old chromosome preparations according to the protocol described in Rosato and Rosselló (2009).

### DNA probe preparation

The clone pTa71, 9 kb EcoRI fragment containing the 18S–5.8S–26S rDNA genes and the intergenic spacer regions from *Triticum aestivum* L. (Gerlach and Bedbrook 1979), was used as heterologous probe of the 45S rDNA loci. The pTa794 probe, a cloned 410 bp BamHI fragment of the 5S rDNA, including the 120 bp gene and the intergenic spacer isolated from *T. aestivum* (Gerlach and Dyer 1980), was used as a heterologous probe for the detection of the 5S rDNA loci. Digoxigenin-11-dUTP and Biotin-16-dUTP were used to label pTa71 and pTa794, respectively, by nick translation according to the manufacturer's protocols (Roche, Germany). Both clones were used as probe for *in situ* hybridization on metaphase chromosome spreads and interphase nuclei.

### *In situ* hybridization

Fluorescent *in situ* hybridization (FISH) protocols were carried out according to Rosato *et al*. (2008) except for one additional stringent wash following the hybridization at 37 °C in 1× saline sodium citrate for 30 min. Chromosomes were mounted in Vectashield antifade (Vector Laboratories) containing 5 µg mL^−1^ of 4',6-Diamidino-2-phenylindole (DAPI) as counterstain. Hybridization signals were analysed using an epifluorescence Olympus microscope, with appropriate filter set, equipped with an Olympus Camedia C-2000-Z digital camera. The images were optimized for best contrast and brightness by image processing software (Adobe Photoshop v. 7.0).

### Chromosome number evolution analysis

In order to infer patterns of cytogenetic evolution in Vellinae, our cytogenetic results have been integrated in a phylogenetic framework. To determine the direction of chromosomal change, three approaches were followed. First, the software ChromEvol 2.0 (Mayrose *et al*. 2010) was used to infer the chromosome number evolution model and haploid ancestral chromosome numbers by maximum likelihood (ML) and Bayesian methods. To choose the best model among the eight models provided by the programme, the ‘All-Models’ option was used. The maximum number of chromosomes was set to two times the highest number found in the empirical data, and the minimum number was set to 2. The number of simulations was 10 000 and the one that best fit the data set was selected under the Akaike information criterion (AIC). Second, mapping of cytogenetic features onto a phylogenetic framework was also carried out following the likelihood reconstruction method in Mesquite version 2.7 (Maddison and Maddison 2009), assigning to each ancestral node the state that maximizes the probability of obtaining the observed states in the terminal taxa under the specified model of evolution (Mk1 model, in this study). Third, the most parsimonious reconstruction of the ancestral character states for chromosome number and number of rDNA sites were estimated using MacClade version 3.0 (Maddison and Maddison 1992).

The molecular phylogenetic tree to be used as an evolutionary framework for Vellinae was constructed from newly generated sequences from *V. pseudocytisus* subsp. *orcensis*, and previously published nuclear ribosomal ITS sequences (Crespo *et al*. 2000, 2005) that were downloaded from GenBank (Table 1) and aligned with the sequence editor utility of DAMBE software (Xia and Xie 2001). *Succowia balearica*, which was shown to be basal in Vellinae (Crespo *et al*. 2000, 2005), was used as an outgroup. Maximum likelihood analyses were conducted using the ML method implemented in MEGA version 5.1 (Tamura *et al*. 2011; available at http:www.megasoftware.net) using the best substitution model for each data set according to BIC and AIC. Initial trees for the heuristic search were obtained automatically by applying Neighbor-Join and BioNJ algorithms to a matrix of pairwise distances estimated using the Maximum Composite Likelihood approach, followed by the selection of the topology with the highest likelihood value. The ML bootstrap values were based on 100 replicates.

## Results

### Chromosome numbers

Chromosome numbers observed at mitotic metaphase cells of all species from Vellinae are reported in Table 2. Chromosome counts vary from 2*n* = 16 (*Carrichtera annua*) to 2*n* = 136 (*V. lucentina*). Chromosome numbers of *V. castriliensis* (2*n* = 102) and *V. lucentina* (2*n* = 136) found in this study disagree with previous counts reporting lower numbers (Table 2). Our cytogenetic results in *V. pseudocytisus* subsp. *paui* agree with one of the two earlier counts available (2*n* = 34), but the 2*n* = 68 cytotype could not be verified.

**Table 2.** Karyotypic features of Vellinae. The chromosome number, ploidy level (x), total number of 45S and 5S rDNA loci, maximum number of nucleoli and distribution of 45S and 5S rDNA loci in each rDNA-bearing chromosomal type are indicated. ^1^Maire (1967); ^2^Crespo *et al.* (2005); ^3^Crespo (1992); ^4^Domínguez Lozano *et al*. (2003).

Taxon	2*n*	Ploidy level	Deviant chromosome counts	45S rDNA loci	5S rDNA loci	Max. no. nucleoli	I 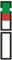	V 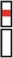	VI/VII 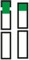
*Succowia balearica*	36	4x	32^1^	2	2	2	–	2	2
*Carrichtera annua*	16	2x	–	2	1	2	–	1	2
*Vella anremerica*	34	2x	–	2	2	2	–	2	2
*V. aspera*	102	6x	–	6	2	–	–	2	6
*V. bourgeana*	34	2x		2	1	2	–	1	2
*V. castrilensis*	102	6x	68^2^	9	4	3	2	2	7
*V. lucentina*	136	8x	34^3^	21	8	13	3	5	18
*V. mairei*	68	4x	–	5	4	–	–	4	5
*V. pseudocytisus*									
subsp. *pseudocytisus*	68	4x	–	6	3	6	1	2	5
subsp. *glabrata*	34	2x	–	4	1	4	–	1	4
subsp. *orcensis*	34	2x	–	3	2	6	1	1	2
subsp*. paui*	34	2x	68^4^	2	2	–	–	2	2
*V. spinosa*	34	2x	–	1	2	–	–	2	1

### Reconstruction of ancestral chromosome numbers

**Figure 2.**
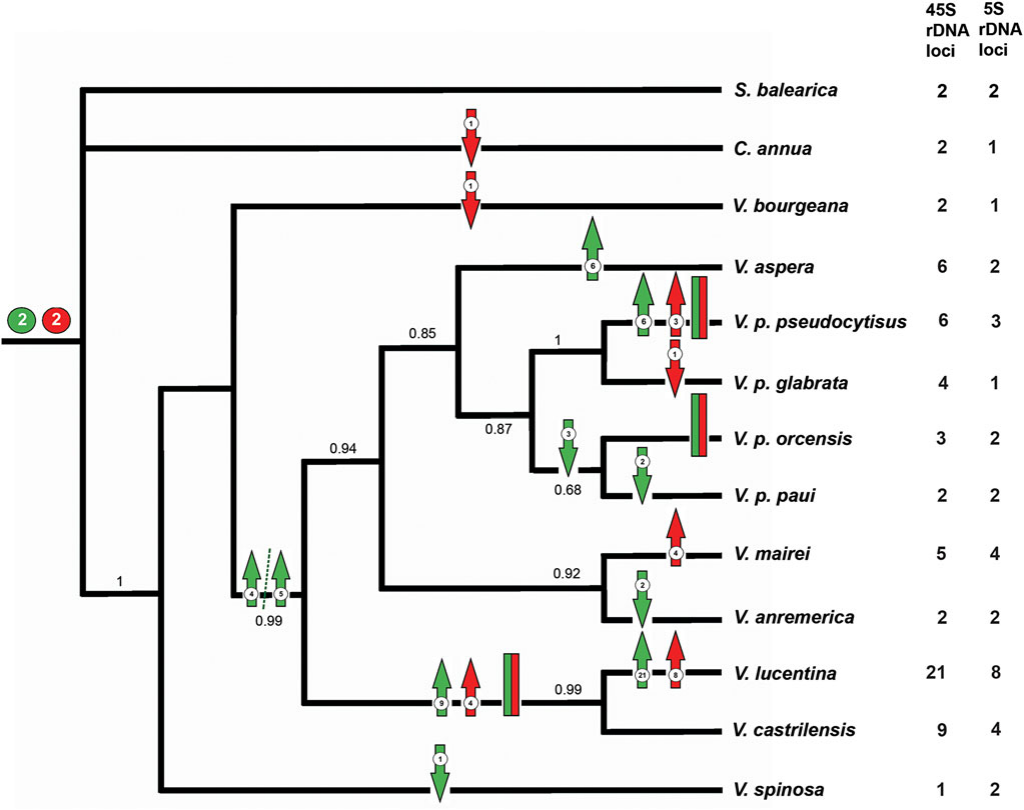
State assignments of 45S and 5S rDNA loci number on the ML tree based on nuclear ribosomal ITS sequence data for Vellinae. Bootstrap percentages, based on 100 replicate analyses, are shown above nodes. The inferred evolutionary events implying loss or amplification of rDNA sites and the chromosomal linkage of 45S and 5S rDNA loci are shown. Upward-pointing and downward-pointing arrows indicate locus gains and locus loss, respectively. Parsimony reconstruction of the ancestral 45S rDNA loci number in the node of the core *Vella* is four or five. The red and green bars represent the chromosomal linkage of 5S and 45S rDNA loci, respectively.

To describe inferred chromosome evolution in Vellinae, we focus on the Bayesian inference based on ITS sequences depicted in Fig. 2. The best supported model in ChromEvol software infers polyploidization and demipolyploidization as main processes in chromosome evolution occurring at 10 and 3 nodes, respectively (Table 3). Two single gains in chromosome number were inferred in *Succowia* and at the shared ancestor between *Carrichtera* and *Vella*, while no chromosomal losses were hypothesized in the model.

**Table 3.** Results from the eight models analysed to infer chromosome number evolution in Vellinae under the ChromEvol package (Mayrose *et al*. 2010). Columns indicate model name (CR, Constant_Rate; CRD, Constant_Rate_Demi; CRDE, Constant_Rate_Demi_Est; CRND, Constant_Rate_No_Duplication; LR, Linear_Rate; LRD, Linear_Rate_Demi; LRDE, Linear_Rate_Demi_Est; LRND, Linear_Rate_No_Duplication); LogLiK, logarithmic likelihood; AIC, Akaike information criterion; rate parameters (*λ*, chromosome gains; *δ*, chromosome losses; *ρ*, polyploidy; μ, demipolyploidy; *λ*_1_/*δ*_1_, gains and losses depending linearly on the current chromosome number); frequency of the four possible event types with a posterior probability (PP) > 0.5; best haploid chromosome numbers inferred at the root node under Bayesian optimization with the respective PP and under ML.

Model	LogLik	AIC	*λ*	*δ*	*Ρ*	*μ*	*λ*_1_	*δ*_1_	Events inferred with PP > 0.5				Chromosome no. at Vellinae root node		
									Gains	Losses	Dupl.	Demi.	Bayes: best *n*; PP	Bayes: second best *n*; PP	ML
CR	−53.2	112.6	43.6	48.4	2.2	–	–	–	263.8	272.2	28.3	0	1; 0.11	2; 0.1	1
CRD	−28.6	63.3	0.1	≪0.01	1	1	–	–	1.7	0	8.6	4	8; 0.76	4; 0.16	8
CRDE	−27.5	63	0.2	≪0.01	1.8	0.6	–	–	2.4	0	10.8	3.3	8; 0.6	4; 0.33	8
CRND	−76.7	157.4	87.6	80.2	–	–	–	–	518.9	454.9	12.9	0	7; 0.07	8; 0.07	6
LR	−49.6	109.4	5.9	≪0.01	2.3	–	1.05	0.3	67.5	93.5	12.7	0	6; 0.13	5; 0.12	4
LRD	−28.7	67.3	0	≪0.01	1	1	≪0.01	0.01	1.3	0	8.3	3.9	8; 0.84	4; 0.13	8
LRDE	−27.5	67.1	0.1	≪0.01	1.8	0.6	≪0.01	0.004	2.3	0	10.8	3.4	8; 0.6	4; 0.34	8
LRND	−56	120.1	67.4	80	–	–	2.01	4.24	744.8	601.7	36.6	0	1; 0.18	2; 0.14	1

### Nuclear rDNA loci

**Figure 3.**
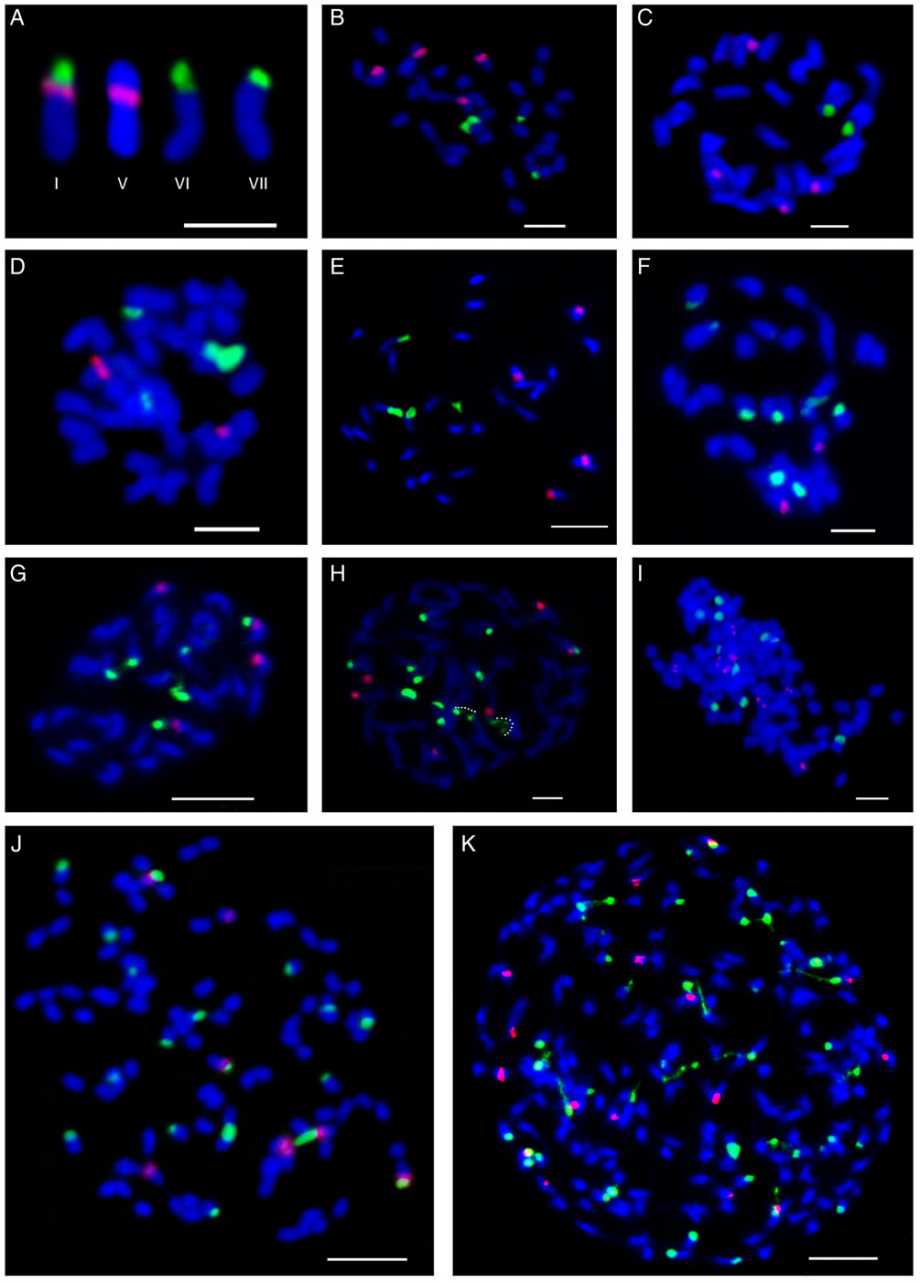
Physical mapping of rDNA loci in subtribe Vellinae showing the localization of 45S rDNA (green) and 5S rDNA (red) loci on somatic chromosomes counterstained with DAPI (blue). (A) Identification of chromosome landmarks (I–VII) bearing 45S and 5S sites found in Vellinae. (B) *Succowia balearica* (2*n* = 36). (C) *Vella spinosa* (2*n* = 34). (D) *Vella bourgeana* (2*n* = 34). (E) *Vella pseudocytisus* subsp. *paui* (2*n* = 34). (F) *Vella pseudocytisus* subsp. *glabrata* (2*n* = 34). (G) *Vella pseudocytisus* subsp. *orcensis* (2*n* = 34). (H) *Vella pseudocytisus* subsp. *pseudocytisus* (2*n* = 68). Decondensed signals belonging to the same 45S rDNA site are linked by white dots. (I) *Vella mairei* (2*n* = 68). (J) *Vella castrilensis* (2*n* = 102). (K) *Vella lucentina* (2*n* = 136). Scale bar: A = 5 μm; B–K = 10 μm.

**Figure 4.**
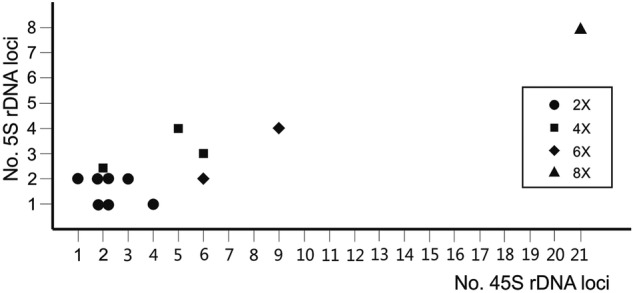
Bivariate plot showing the relationship between the number of 45S and 5S rDNA loci in diploid and polyploid Vellinae taxa.

Simultaneous FISH localization of the 45S and 5S rDNA multigene families were assessed for the first time in all Vellinae taxa (except in *C. annua* where the number of 45S rDNA loci were previously known; cf. Ali *et al*. 2005) and are summarized in Table 2. The variation found in the number of 45S rDNA loci in subtribe Vellinae was considerable, and ranged from 2 sites in *V. spinosa* to 42 sites in *V. lucentina* (Fig. 3). The monotypic genera *Succowia* and *Carrichtera* showed the same number of loci (two) despite the fact that the former is tetraploid and the later diploid (Table 2). Lack of a strict correspondence between ploidy level and number of 45S rDNA loci was also observed in *Vella* (Fig. 4, Table 2). The diploids *V. anremerica*, *V. bourgeana* and *V. pseudocytisus* subsp. *paui* showed two 45S rDNA loci, double the number present in *V. spinosa*. In contrast, *V. pseudocytisus* subsp. *orcensis* and *V. pseudocytisus* subsp. *glabrata* showed three and four loci, respectively. The tetraploids *V. mairei* and *V. pseudocytisus* subsp*. pseudocytisus* showed five and six loci, respectively. Meanwhile, the two hexaploid species also differed, showing six loci *V. aspera* and nine loci *V. castrilensis*. The octoploid *V. lucentina*, with 21 loci, showed the highest number of 45S rDNA loci recorded to date not only in Vellinae but also in the whole Brassicaceae.

Ag-NOR staining performed in a subset of the sampled taxa (Table 2) revealed that in diploid and tetraploids, all 45S rDNA loci appear to be active, as the number of sites equate the maximum number of nucleoli detected in interphase nuclei. In contrast, silencing of between 33 and 61 % rDNA loci in higher polyploids (hexaploids and octoploids) was inferred.

The range of variation in 5S rDNA loci number observed was narrower than that in the 45S multigene family. Thus, diploids show one or two loci and tetraploid species or cytotypes, three or four loci. The number of loci in hexaploid and octoploids ranges from two (*V. aspera*) to eight (*V. lucentina*).

The chromosomes of Vellinae are small and showed an overall similar morphology, precluding the building of accurate idiograms where the rDNA loci could be mapped. However, it was possible to describe the rDNA chromosomal localization according to the chromosome type bearing 45S rDNA and 5S rDNA loci. These chromosome landmarks, numbered I–VIII, were described by Hasterok *et al*. (2001, 2006) for understanding the relationship and the evolution of polyploidy in species of Brassicaceae.

Three landmark chromosomal types showing rDNA genes were observed in Vellinae (Table 2; Fig. 3A). Type I includes chromosomes bearing a terminal 45S rDNA site that is adjacent to the 5S rDNA site on the same arm. The chromosome type V shows a 5S rDNA site on the short arm that is located in an interstitial position. Finally, the chromosome type VI (including type VII) was characterized by the presence of a terminal site of 45S rDNA on the short arm. Chromosome types VI and VII were subsumed within a single group, as the discriminating features indicated by Hasterok *et al.* (2001) were considered to be not reliable. According to these authors, chromosome types VI and VII were distinguished by the distension or non-distension of the secondary constriction, respectively.

Chromosome type I showed a restricted distribution within Vellinae, being absent in *Carrichtera* and *Succowia*, and it was exclusively observed in *V. pseudocytisus* (subsp. *pseudocytisus* and subsp. *orcensis*), in *V. castrilensis* and in the high polyploid *V. lucentina* (Table 2). In contrast, type V, VI and VII chromosomes were identified in all accessions.

### Ribosomal DNA changes in a phylogenetic framework

Changes of 45S rDNA and 5S rDNA loci, and the acquisition of 45S-5S rDNA collinearity (chromosome type I) based on ML and parsimony estimates are shown on the ITS phylogenetic tree shown in Fig. 2. Our phylogenetic tree is congruent with phylogenies constructed using this marker (Crespo *et al*. 2000; Simón-Porcar *et al*. 2015). Two 45S rDNA loci were inferred to be basal in Vellinae, and a single event of rDNA loss was inferred for the *V. spinosa* lineage. Bursts of rDNA amplification appeared independently in all clades after the split of *V. bourgeana*, involving both diploid and polyploid lineages. The pattern of 5S rDNA evolution revealed a contrasting history. 5S rDNA loci losses from a two-locus ancestral state were inferred to have occurred independently three times near the root node: in *C. annua*, in *V. bourgeana* and in a single lineage of *V. pseudocytisus* (subsp. *glabrata*). Ribosomal DNA stasis in 5S loci number was inferred for most internal nodes, whereas loci gain accompanied high polyploidization events were inferred in the *V. lucentina*–*V. castrilensis* clade, and independently in the polyploids *V. pseudocytisus* subsp. *pseudocytisus* and *V. mairei*.

The 45S-5S rDNA linkage apparently appeared before the split of the *V. lucentina*–*V. castrilensis* clade and independently in two lineages of *V. pseudocytisus* (subsp. *pseudocytisus* and subsp. *orcensis*).

## Discussion

### Chromosome numbers

With the exception of four *Vellinae* species, chromosome counts and ploidy levels detected in this study agree with reports published in previous works (Table 2). Discordant results were found for the narrowly distributed *V. lucentina* (2*n* = 136), previously reported to have 2*n* = 34 (Crespo 1992). Our mitotic chromosome counts were verified in >20 individuals from two accessions, and corroborated by the additional observation of 68 bivalents at meiotic I prophase meiocytes (data not shown), confirming the sporophytic complement obtained from root tips. Intriguingly, one of the accessions studied comes from the type locality where the chromosome number of 2*n* = 34 was reported (Crespo 1992). For *V. castriliensis*, from which a very small population is known, in this study, we report 2*n* = 102 and we cannot support the 2*n* = 68 chromosome number reported for this species in Crespo *et al*. (2005).

The reasons for these contrasting ploidy levels may be biological (e.g. true intraspecific cytotypes, intraindividual endopolyploidy) or artefactual, linked to poorly preserved material or erroneous observations. To date, intrapopulational variability regarding chromosome number has not been reported in Vellinae and we have not found it in this study either. Since previous cytogenetic results on *V. lucentina* and *V. castriliensis* were not vouched by micrographs or diagrams (Crespo 1992; Crespo *et al*. 2005), we cannot consider them as sound evidence of chromosomal polymorphism in these species and thus have not been included in our analyses.

Our results on *V. pseudocytisus* subsp. *paui* (2*n* = 34) agree with the original report of Gómez-Campo (1981). Domínguez Lozano *et al*. (2003) erroneously attributed the count 2*n* = 68 to this subspecies (F. Domínguez Lozano, pers. comm.), a misunderstanding that was echoed by Pérez-Collazos and Catalán (2006, 2011). These latter authors rejected the diploid 2*n* = 34 count on the basis that their isozyme data (gene duplication and fixed heterozygosity) best supported a tetraploid status for this subspecies (2*n* = 68). However, the complex isozyme profiles detected by Pérez-Collazos and Catalán (2006, 2011) have also been detected in other putative diploids from Brassiceae (e.g. *Parolinia*; Fernández-Palacios *et al*. 2006) and may be due to, and account for, the several paleoduplication events produced along Brassicaceae evolution (Ziolkowski *et al*. 2006; Lysak *et al*. 2007).

Our cytogenetic survey suggests more complex patterns of polyploid evolution than previously noted for Vellinae, in which *V. aspera* (2*n* = 102; 6x) was considered to be the evolutionary culmination of a single polyploid series (2x, 4x and 6x) originated in *V. pseudoctysus* and showing a clinal correlation with geography (Fig. 1; Pérez-Collazos and Catalán 2006, 2011). According to our study, high polyploidization events (6x, 8x) arose independently in the more basal clade *V. castrilensis*–*V. lucentina*, where extant diploid species are unknown, and for which ploidy level and altitude do not appear to be related as previously suggested (Gómez-Campo 1981).

The chromosome number found in *V. lucentina* (2*n* = 136) constitutes the highest number reported for the tribe Brassiceae, and it is among the highest records known to date for the whole Brassicaceae family (together with some *Cardamine* and *Crambe* L. species; Warwick and Al-Shehbaz 2006; Franzke *et al*. 2011; Lysak and Koch 2011).

### Karyological evolution

Best-fitting model of chromosome number evolution with a high likelihood score suggests that the Vellinae core showing *x* = 17 chromosomes arose by duplication events from a recent *x* = 8 ancestor shared by *C. annua*. Fine molecular cytogenetic work has determined a putative Ancestral Karyotype of Brassiceae (*n* = 8) with 24 conserved genomic blocks (Lysak *et al*. 2005, 2006; Schranz *et al*. 2006), providing compelling evidence of an ancient hexaploidization event in the tribe, to which Vellinae belongs. High karyotypic dynamism and genomic rearrangements leading to (descending) changes in the chromosome number have been postulated, resulting in genome diploidization (Lysak *et al*. 2005).

Mapping of two ancestral cruciferous blocks F and L identified six copies in a 2*n* = 68 accession of *V. pseudocytisus* s.l. (Lysak *et al*. 2007; no infraspecific identification was provided). These observations suggest that *Vella* species showing 2*n* = 34 did not undergo further polyploid events after Brassiceae diversification and shared the same paleoploidy level as the primary hexaploid ancestor of the tribe. Data based on isozyme number variation in Brassiceae agree with this view, suggesting that genera showing *n* = 14–18 did not arise from polyploidy of the *n* = 7–13 genomes (Anderson and Warwick 1999).

Thus, results from isozyme number in *V. bourgeana*, *V. anremerica* and *V. pseudocytisus* (2*n* = 34 and 2*n* = 68 cytotypes) strongly support that the *x* = 17 base chromosome number in Vellinae evolved by aneuploidy and/or chromosome rearrangements rather than allopolyploidy from *x* = 8 and *x* = 9 genomes as suggested earlier on intuitive grounds (Manton 1932; Gómez-Campo 1981; Crespo *et al*. 2000).

Overall, total evidence from molecular cytogenetics and isozyme data strongly suggests that ancestral karyotype of the core Vellinae was hexaploid and that two divergent pathways of chromosome evolution were followed: (i) homoploid rearrangements leading to a low chromosome number in *Carrichtera* and (ii) recurrent and independent neoploidization events occurring during the diversification of *Vella* lineages. Model-based methods of inferring chromosome evolution contradict the first hypothesis, although support the highest chromosome numbers found in *Vella* as being derived and not ancestral, in agreement with the second hypothesis.

The neopolyploid origin of the core Vellinae is supported only by a likelihood-based method that takes into account a single parameter (chromosome number) without incorporating solid knowledge on karyological evolution posited during Brassicaceae evolution. This raises the question whether firm hypothesis on karyological evolution based on total evidence, although not resulting from model-based approaches and therefore statistically not tested, should be given more credibility than likelihood-based methods performed on evolutionary crude data, as chromosome number alone. The Brassicaceae, where a wide and solid molecular and karyological knowledge has been accumulated, could be an excellent case study to further evaluate the goodness of fit of modelling chromosome evolution in a highly complex scenario.

### Trends in site-number change of rDNA in Vellinae

Although comparative mapping and genomics in Brassicaceae (including tribe Brassiceae) have postulated a unified syntenic framework for conserved blocks of crucifer genomes (Schranz *et al*. 2006), no specific hypothesis on the ancestral distribution of rDNA loci could be assumed (Mandáková and Lysak 2008). Thus, claims supporting the hexaploidization event in tribe Brassiceae (and hence the ancestral state) by the presence of three 45S rDNA loci in some species (Anderson and Warwick 1999) seem unfounded given the frequent mobility of rDNA sites.

In fact, reconstruction of ancestral rDNA states in Vellinae in this study supports the inference that the ancestral number of loci in the subtribe was two for each multigene family. This suggests that even if a conservative minimum number of one loci of 45S and 5S rDNA is estimated to be present in the ancestor species predating the hexaploidization event in tribe Brassiceae, an overall tendency towards a net loss of rDNA loci (perhaps not lineal) occurred during the splitting of Vellinae ancestors from the remaining Brassiceae lineages. However, a contrasting pattern for 45S rDNA and 5S rDNA site change in both paleopolyploid and neopolyploid species was linked to diversification of Vellinae lineages, suggesting dynamic and independent changes in rDNA site number during speciation processes and a significant lack of correlation between 45S and 5S rDNA evolutionary pathways.

Thus, only losses of 5S rDNA loci are inferred during homoploid evolution in Vellinae, but trends for 45S rDNA changes involved gains and losses of sites. Interestingly, the number of 5S rDNA loci are always equal or lower than expected in polyploid taxa, i.e. losses of up to four loci in *V. aspera* (6x), two loci in *V. castrilensis* (6x) and one loci in *V. pseudocytisus* (4x) have been inferred (Fig. 2). This apparently suggests not only the existence of constrictions to burst loci amplification in the 5S rDNA multigene family in polyploids, but also an overall trend to further reduce their number, at least more frequently than in the diploid taxa.

The 45S rDNA site change in polyploids tells a different story, implying loci amplification in four out of six polyploid entities. Specifically, the case of the octoploid *V. lucentina* is particularly relevant, as a drastic increase in 45S rDNA loci number (21 instead of the 8 expected) was linked to its evolutionary history. Available evidence from further polyploid complexes in Brassiceae suggests the predominance of species harbouring labile 45S loci prone to amplification across chromosomes (Garcia *et al*. 2012; Zozomová-Lihová *et al*. 2014).

To what extent these inferred changes in rDNA site evolution in Vellinae are paralleled in other Brassicaceae lineages and reflect similar evolutionary trends is not easy to assess. First, and despite valuable contributions, knowledge on rDNA site-number variation within the family is still fragmentary and manifestly incomplete for most monophyletic lineages to be meaningful (see Ali *et al*. 2005; Hasterok *et al*. 2006 for major contributions). Second, the most extensive sampling has been focussed on crop species, where modifications in their rDNA genome organization may have occurred as a result of intense artificial selection (e.g. Pedrosa-Harand *et al*. 2006). Third, and most importantly, available rDNA data in Brassicaceae have not been discussed against phylogenetic inferences, precluding the building of solid hypotheses about patterns of rDNA site change. Even so, data obtained for most core genera of the tribe Brassiceae (e.g. *Brassica*, *Sinapis*, *Diplotaxis* and *Erucastrum*) are difficult to put into evolutionary perspective, as many of them are manifestly polyphyletic (Warwick and Sauder 2005), which implies that more data from other lineages should be gathered.

## Conclusions

Our finding that changes in 45S and 5S rDNA sites are decoupled through *Vellinae* evolution is intriguing, as an stoichiometric relationship of mature rRNA copies from genes of both loci is required for ribosome biogenesis (Fromont-Racine *et al*. 2003). The most obvious explanation is that differential patterns of RNA transcription may exist in the two rDNA families to overcome the presence of extra copies of rRNA species in the cytoplasm, where ribosome subunits are assembled (Fromont-Racine *et al*. 2003). However, knowledge concerning rDNA copy number in Vellinae (which is totally absent) should be gained in order to assess whether changes in rDNA sites present covariation with rDNA gene number.

## Sources of Funding

This research was supported by funds from the Spanish Ministry of Education and Science (Project CGL2010-22347-C02-01), the Catalan Government (Consolidated Research Group 2009SGR608) and by a Ph.D. grant from the Spanish Ministry of Education and Science to J.A.G.

## Contributions by the Authors

All authors conceived the idea and sampled accessions. M.R. collected the cytogenetic data, J.C.M.-S. and J.A.G. performed the phylogenetic analyses. J.A.R. led the writing with assistance from the others. All authors edited manuscript drafts and responded to reviewer comments.

## Conflict of Interest Statement

None declared.
